# Supplementary material for: Nutritional Content, Labelling and Marketing of Breakfast Cereals on the Belgian Market and Their Reformulation in Anticipation of the Implementation of the Nutri-Score Front-Of-Pack Labelling System
Source: Nutrients. 2020 Mar 25;12(4):884. doi: 10.3390/nu12040884 (PMC7230794; doi:10.3390/nu12040884)
Supplement: Supplementary file 1 [file nutrients-12-00884-s001.pdf]

Supplementary material 1

Claim types

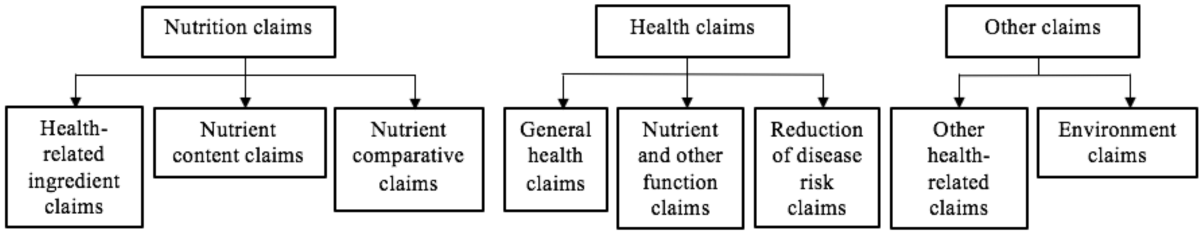

Figure 1 Categories and sub-categories of each claim type as per the taxonomy of the International Network for Food and Obesity/NCDs Research, Monitoring and Action Support (INFORMAS)

Claim Content - examples

Health-related ingredient claims

- Wholegrain
- Fruits/nuts/honey
- Grains/seeds
- Vegetables/plants
- Bacteria/culture/probiotics/prebiotics
- Edible oils/oil emulsions
- Cocoa/cacao
- Water

Nutrient content claims

- Fibre
- Energy/calories
- Antioxidants/vitamins/minerals/hormones
- Carbohydrates
- Fats
- Sugar
- Protein
- Salt

Nutrient comparative claims

- Reduced fat
- More calcium
- Less salt
- Reduced sugar
- Reduced calories
- More fibre
- Reduced carbohydrates
- More protein
- More vitamins/minerals/hormones/antioxidants

General health claims

- General e.g. super, healthy
- Low GI/energy density/lower GI
- Digestive health
- Bone health
- Oral health
- Immune health

Nutrient and other function claims

- Nutrient + muscle
- Nutrient + bone
- Nutrient + growth
- Nutrient + vision
- Nutrient + energy
- Nutrient + strength
- Nutrient + brain
- Nutrient + nutrient absorption/production
- Nutrient + digestion/bowel
- Nutrient + immunity
- Nutrient + overall health
- Nutrient + blood-related
- Nutrient + oral health

Reduction of disease claims

- Heart-related
- Heart Foundation Tick
- Cholesterol absorption
- Glycaemic impact
- Osteoporosis

- Digestive health
- Nutrient absorption

Other claims

- Environmental
- Other health-related

<u>Glossary</u>

(Adapted from: Rayner M, Wood A, Lawrence M, Mhurchu CN, Albert J, Barquera S, et al. Monitoring the health- related labelling of foods and non-alcoholic beverages in retail settings. Obes Rev. 2013;14(Suppl. 1):70-81)

Nutrition claim: any representation which states, suggests or implies that a food has particular nutritional properties including but not limited to the energy value and to the content of protein, fat and carbohydrates, as well as the content of vitamins and minerals.

Health-related ingredient claim – any representation which states, suggests or implies that a food has particular nutritional properties not related to its energy value or to the content of protein, fat and carbohydrates, vitamins and minerals but related to the content of an ingredient'

Nutrient content claim: ‘a nutrition claim that describes the level of a nutrient contained in a food [or its energy value; includes ‘non-addition claims’ - ‘any claim that a [nutrient] has not been added to a food, either directly or indirectly. The [nutrient] is one whose presence or addition is permitted in the food and which consumers would normally expect to find in the food’

Nutrient comparative claim: ‘a [nutrition] claim that compares the nutrient levels and/or energy value of two or more foods.’

Health claim: ‘any representation that states, suggests, or implies that a relationship exists between a food or a constituent of that food and health.’

Nutrient function claim – ‘a [health] claim that describes the physiological role of the nutrient in growth, development and functions of the body.’ (CAC/GL 23-1997) [Although Codex classifies nutrient function claims as nutrition claims it seems more logical to classify them as health claims]

Other function claim – health ‘claims concerning specific beneficial effects of the consumption of foods or their constituents, in the context of the total diet on normal functions or biological activities of the body. Such claims relate to a positive contribution to health or to the improvement of a function or to modifying or preserving health.’

Reduction of disease risk claim – health ‘claims relating the consumption of a food or food constituent, in the context of the total diet, to the reduced risk of developing a disease or health-related condition.’

General health claim – a health claim concerning the general beneficial effects of the consumption of foods or their constituents on health.
